# Detailed pedigree analyses and prenatal diagnosis for a family with mucopolysaccharidosis type II

**DOI:** 10.1186/s12920-021-01027-5

**Published:** 2021-06-30

**Authors:** Chuan Zhang, Shengju Hao, ZhaoYan Meng, Ling Hui, Yan Wang, Feng Xuan, Xue Chen, Xing Wang, Furong Zheng, Lei Zheng, Bingbo Zhou, Xinqi Wu, Qinghua Zhang, Zongfu Cao

**Affiliations:** 1 Gansu Province Maternal and Child Health Care Hospital, Lanzhou, 730050 China; 2 National Research Institute for Health and Family Planning, National Human Genetic Resources Center, Graduate School of Peking Union Medical College, Beijing, 100081 China

**Keywords:** MPS II, *IDS*, Developmental delay, Special face, Skeletal malformation

## Abstract

**Background:**

Mucopolysaccharidosis type II (MPS II) is an X-linked multisystem disorder caused by mutations in the gene encoding iduronate 2-sulfatase (*IDS*). The clinical manifestations of MPS II include skeletal deformities, airway obstruction, cardiomyopathy, and neurologic deterioration. MPS II has high genetic heterogeneity disorder, and ~ 658 variants of *IDS* have been reported.

**Methods:**

We undertook a detailed pedigree analysis of four patients within the same family by targeted next-generation sequencing and Sanger sequencing.

**Results:**

We identified a novel heterozygous frameshift variant, c.1224delC(p.Pro408ProfsTer31), of *IDS* in three patients. We defined c.1224delC as a pathogenic variant according to the 2015 guidelines set by the American College of Medical Genetics and Genomics.

**Conclusion:**

We reported the second Chinese female MPS II patient. We helped to ensure that these two families had healthy babies. Our findings have enlarged the mutational spectrum of *IDS*, and these findings could be useful for genetic counseling and the prenatal diagnosis of MPS II.

## Introduction

Mucopolysaccharidosis II (MPS II; Online Mendelian Inheritance in Man number (OMIM#): 309900) is a rare X-linked recessive disorder. MPS II leads to progressive accumulation of glycosaminoglycans in nearly all cell types, tissues, and organs. MPS II has an incidence of about 0.38–1.09 per 100,000 population, and is the only X-linked-inherited MPS [1, 2].

The clinical symptoms of MPS II vary. MPS II can be divided into two main forms: “attenuated” and “severe” [2, 3]. Patients suffering from MPS II often appear normal at birth, and clinical characteristics usually appear between 2 and 4 years of age. In general, the severe form of MPS II presents much earlier. The attenuated form of MPS II often has a slow progression of symptoms [2]. Progressive cognitive deterioration or neurologic involvement are the main factors that determine the severity of MPS II, and ~ 75% of patients have the severe type [3–5].

The clinical manifestations of MPS II include severe obstruction of the airways, skeletal deformities, cardiomyopathy, and, in most patients, neurologic decline. Death usually occurs by the age of 20 years but some patients with less severe disease can live to 50–60 years [4].

MPS II is caused by a mutation in the gene encoding iduronate 2-sulfatase (IDS; OMIM#: 300823) on chromosome Xq28. MPS II has high genetic heterogeneity. To date, 658 variants of *IDS* have been reported, including missense/nonsense variants, small deletions, splicing variants, and frameshift variants [2].

We undertook a detailed pedigree analysis of four patients within the same family. We identified a novel frameshift variant, c.1224delC, of *IDS* in these patients. We provided an invasive prenatal diagnosis for two families with this pedigree, and helped to ensure that healthy babies were delivered.

## Materials and methods

This study was undertaken according with the tenets of the Declaration of Helsinki 1975 and its later amendments. The study protocol was approved by the Ethics Committee of Gansu Provincial Maternal and Child Health Care Hospital (Gansu, China). Written informed consent was obtained from all participants or their legal guardians who participated in this study.

### Patients

**Fig. 1:**
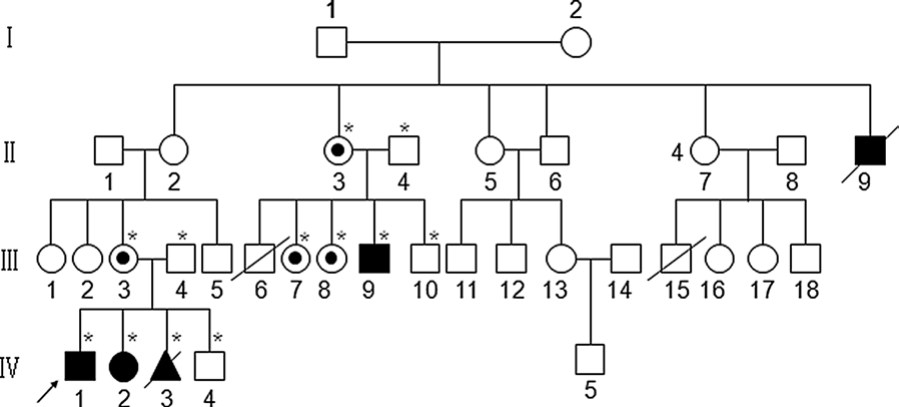
The pedigree of this family. The available samples are marked as ∗ satiric symbol

**Fig. 2:**
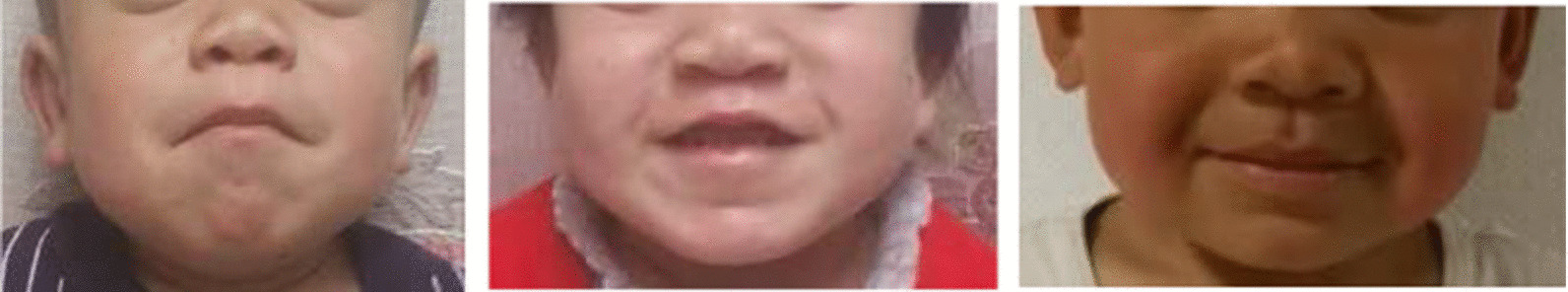
The patient's dysmorphic facial features—broad nasal bridge, large rounded cheeks, and thick large lips

There were four patients in this family: II9, III9, IV1 and IV2 (Fig. 1). II9 died at 32 years of age owing to cardiorespiratory disease. The proband was IV1; at the age of 3 years, due to developmental delay as well as face/skeletal malformations (Fig. 2), IV1 was referred to our Medical Genetics Center for genetic counseling in 2015. He has a sister with similar clinical characteristics. His mother was 12-weeks pregnant at the time, and wanted a genetic test and a prenatal diagnosis. We undertook a urinary glycosaminoglycans (uGAGs) test and molecular diagnosis for the proband. The result for the uGAGs test was positive, and a molecular test diagnosed the proband as having MPS II. The mother took an invasive prenatal diagnosis at 20 weeks of pregnancy: the male fetus had the same mutation site as that of the proband, and she chose to terminate the pregnancy.

In 2017, we undertook the second invasive prenatal diagnosis for III3. This time, a pathogenic variant was not detected in the male fetus. She delivered this fetus and, at present, the boy is healthy.

In 2019, II3 and II4 came to our Medical Genetics Center for genetic counseling. They also have a child with MPS II. The facial features of the child were normal before the age of 2 years, but began to develop the features of MPS II after that age. II3 is 19-weeks pregnant, and we have provided an invasive prenatal diagnosis for her. The fetus is male and the *IDS* variant has not been detected in him.

### uGAG analyses and biochemical analysis

The morning urine specimen of three patients and the amniotic fluid/urine after the birth of III10 were obtained for GAG analyses. The latter were carried out according to the procedure described by de Jong and colleagues [6]. The urinary level of creatinine was measured using a biochemical reagent kit (Maccura Biotechnology, Beijing, China). IDS activity from a dried blood spot was measured according to the method described by Rezende and colleagues [7].

### Sample collection and preparation of genomic DNA

Blood samples (2–3 mL) were collected from patients and their family members. Genomic DNA was extracted using DNA extraction kit from Tiangen Biotech (Beijing, China).

### Targeted next-generation sequencing (NGS) and Sanger sequencing

**Fig. 3:**
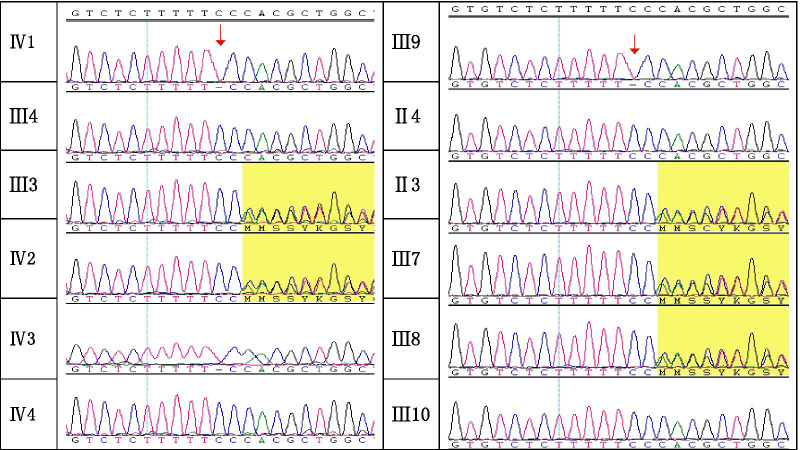
The Sanger sequencing result of c.1224delC(p.Pro408ProfsTer31) on *IDS*

Targeted capture of candidate disease genes was carried out using the GenCap™ Custom Enrichment kit (MyGenostics, Beijing, China). Data analysis and bioinformatics analysis were undertaken according to method described by Zhang and colleagues [8]. The candidate variant c.1224delC of exon 9 of *IDS* was detected in the patients of each family by Sanger sequencing. The primers of exon 9 of *IDS* were 5′-GTTCCTTTACTGCTCCTG-3′ (forward) and 5′-ACATCACATTTGCCATCC-3′ (reverse). The amplification conditions for the polymerase chain reaction for Sanger sequencing were: 95 °C for 5 min, then 20 cycles of 94 °C for 30 s, 62 °C for 45 s, and 72 °C for 1 min, 95 °C for 5 min, then 15 cycles of 94 °C for 30 s, 58 °C for 45 s, and 72 °C for 1 min. DNA sequencing was carried out on an ABI 3500DX Genetic Analyzer (Applied Biosystems, Foster City, CA, USA) (Fig. 3).

### Bioinformatics analysis

The variant was described according to the nomenclature recommended by the Human Genome Variation Society (www.hgvs.org/). Variants were annotated using ANNOVAR (https://annovar.openbioinformatics.org/en/) and filtered according to their predicted effects and allele frequencies in the public database gnomAD (http://gnomad.broadinstitute.org/). Novel variants were checked in the Human Gene Variant Database (HGMD; www.hgmd.cf.ac.uk/), ClinVar database (www.ncbi. nlm.nih.gov/clinvar/) and EXAC database (http://exac.broadinstitute.org/). InterVar (http://wintervar.wglab.org/) was used to evaluate the pathogenicity of all variants according to the standards and guidelines of the American College of Medical Genetics and Genomics [9].

## Results

### Clinical data

All four patients with MPS II had typical clinical features: coarse facial features, growth retardation, skeletal deformities, joint stiffness, umbilical hernias, hepatosplenomegaly, short neck and claw-shaped hands. Specific facial characteristics and umbilical hernias began at the age of 2 years. All patients had neurologic involvement. Magnetic resonance imaging of the cranium of III9 suggested that the patient had white-matter lesions. II9, IV1 and IV2 presented with hearing loss and language loss. II9 was also blind, and II9 died at 32 years of age because of cardiorespiratory failure. At present, III9 does not have neurologic involvement, and has normal hearing and vision.

### Biochemical and molecular analyses

The uGAG/creatinine level (mg/mmol) of III9 was 32.5, whereas it was 79 for IV1 and 99.8 for IV2 (normal value for the age of 1.5–5 years is < 30).The uGAG/creatinine level (mg/mmol) of III10 was 11.2 (amniotic fluid) and 27.2 (in urine 73 h after birth) (normal level for age < 28 days is < 64) [10]. IDS activity from a dried blood spot from patients was 0.91–1.12 μmol/L blood/h (normal cutoff level is > 4.42 μmol/L blood/h) [7].

NGS identified a frameshift variant, c.1224delC (p.Pro408ProfsTer31), of exon 9 in *IDS* for the proband. This result was validated by Sanger sequencing, and this variant was also identified in II3, III3, III7, III8, III9 and IV2.

We provided an invasive prenatal diagnosis for III3 on two occasions, and an invasive prenatal diagnosis for II3 on one occasion. The fetus IV3 of III3 carried the c.1224delC variant, and III3 chose to terminate the pregnancy. The fetus IV4 did not carry this variant, and III3 delivered this male fetus, and this boy is healthy currently. The fetus III10 of II3 is also male and the c.1224delC variant has not been detected in him. This fetus was delivered in March 2020 and he is healthy currently. We told II3 that her two daughters (III7, III8) are carriers of MPS II, and that a prenatal diagnosis will be required if they become pregnant.

### Bioinformatics analysis

The c.1224delC variant has not been reported in HGMD, ClinVar, EXAC or gnomAD databases. According to ACMG criteria, we classified this novel variant as pathogenic (PVS1 + PS2 + PM2 + PP4).

## Discussion

MPS II is caused by a deficiency of the enzyme IDS. The latter is involved in the lysosomal degradation of the GAGs heparan sulfate and dermatan sulfate [11]. *IDS* is located on Xq28, spans 44 kb, and encodes for a polypeptide containing 550 amino acids. *IDS* is a “housekeeping” gene, so MPS II patients may be affected in nearly all cell types, tissues, and organs.

We identified a novel frameshift variant, c.1224delC (p.Pro408ProfsTer31), of *IDS* in the proband (IV1) and other two patients (IV2 of III9) in this family. Although we did not perform functional analysis of this novel variant at the cellular and animal levels, this variant caused a change in amino acids at position 409 and a nonsense mutation at position 439. This action caused the peptide chain to terminate prematurely, which shortened the length of the peptide chain by 111 amino acids. According to ACMG guidelines, we classified this novel variant as a pathogenic variant.

MPS II is an X-linked disorder, but few sporadic cases have been reported in females [12–19]. It has been reported that MPS II is caused in female patients by non-random X-inactivation [2]. There are no “hotspot” variant types in MPS II; most female patients with MPS II carry the maternal variants, whereas only a few cases carry de novo mutations [2]. Sohn et al. reported a female patient with MPS II giving birth to a healthy girl [14]. Her daughter did not show any of the physical signs of MPS II, and the uGAG test was normal. However, because of severe stenosis in pulmonary veins with pulmonary hypertension and a large atrial septal defect, she died at 11 months of age. IV2 of our study was also a female patient, she had the same clinical symptoms as her brother: coarse facial features, growth retardation, skeletal deformities, joint stiffness, umbilical hernias, hepatosplenomegaly, short neck and claw-shaped hands, hearing loss and language loss. She carried the heterozygous variant c.1224delC, and she is only the second Chinese female patient with MPS II reported with this variant. Methylation analyses of specific areas of the X chromosome can detect the status of X-inactivation [14], but, unfortunately, we failed to carry out such analyses. We speculate whether there was a small deletion in the X chromosome of the female patient. Then we performed SNP-array (Affymetrix, CytoScan 750K Array) for the female patient, however, the result of SNP-array was normal, and there was also no LOH at the position of IDS gene (Xq28). Due to limited experimental funds and experimental technology, we did not further explore the cause of the disease. However, according to biochemical results, we thought she was a MPS II patient because her mother carried the same variant and had normal biochemical data.

The clinical symptoms of MPS II include coarse facial features, skeletal deformities and joint stiffness, claw-shaped hands, short stature, cardiorespiratory impairment (including diffuse valvulopathy), inguinal and umbilical hernias, organomegaly, and neurologic involvement [2, 4, 20–23]. Some patients also present with abnormalities in the ear, nose, throat and retina [24]. Among these clinical alterations, cardiorespiratory failure is reported as the most common cause of death [25]. In our study, II9 died at 32 years of age because of cardiorespiratory failure. All four patients had the typical clinical features of MPS II, such as coarse facial features, growth retardation, skeletal deformities, claw-shaped hands, and neurologic involvement. II9, IV1 and IV2 had hearing loss and language loss, and II9 was blind. However, III9 did not present with severe neurologic involvement, and has normal hearing and vision.

MPS II is very rare and there are no hotspot variants in *IDS*, so investigating genotype–phenotype correlations is very difficult. However, large deletions/insertions, nonsense and splicing variants have been reported to be associated with the severe form of MPS II. The variant c.1224delC (p.Pro408ProfsTer31) of *IDS* was identified in our patients, and this frameshift variant eventually led to a nonsense variant. All the patients presented with severe neurologic involvement, and we defined them as having the severe form of MPS II.

A prenatal molecular diagnosis is very important for fetuses at risk of MPS II. In addition, enzymatic analysis can be undertaken on fresh and cultured chorionic villi and cells from amniotic fluid [2]. Undertaking enzymatic analysis and molecular tests simultaneously can increase the reliability of the diagnosis [2, 4, 26]. We provided an invasive prenatal diagnosis for III3 on two occasions and for II3 on one occasion, and helped to ensure that these two families had healthy babies.

In conclusion, we undertook a detailed pedigree analysis of four patients within the same family. A novel frameshift variant, c.1224delC, of *IDS* was identified in these patients. We identified only the second Chinese female patient with this variant. We helped to ensure that these two families had healthy babies. Our findings have enlarged the mutational spectrum of *IDS*, and these findings could be useful for genetic counseling and the prenatal diagnosis of MPS II.

## Data Availability

The datasets generated and/or analysed during the current study are available in the Science Data Bank repository, http://www.scidb.cn/en/s/p3iaAvu.
